# Serum miRNA expression profile as a prognostic biomarker of stage II/III colorectal adenocarcinoma

**DOI:** 10.1038/srep12921

**Published:** 2015-08-07

**Authors:** Jialu Li, Yang Liu, Cheng Wang, Ting Deng, Hongwei Liang, Yifei Wang, Dingzhi Huang, Qian Fan, Xia Wang, Tao Ning, Rui Liu, Chen-Yu Zhang, Ke Zen, Xi Chen, Yi Ba

**Affiliations:** 1 Tianjin Medical University Cancer Institute and Hospital, Key Laboratory of Cancer Prevention and Therapy, Huanhuxi Rd., Tiyuanbei, Tianjin 300060, China; 2 Jiangsu Engineering Research Center for microRNA Biology and Biotechnology, State Key Laboratory of Pharmaceutical Biotechnology, School of Life Sciences, Nanjing University, 22 Hankou Rd., Nanjing 210093, Nanjing, China; 3 Department of Pathology, The Third Xiangya Hospital, Central South University, Changsha, Hunan, China; 4 Department of Clinical Laboratory, Jinling Hospital, Clinical School of Medical College, Nanjing University, Nanjing, China; 5 Department of Neurology, Second Affiliated Hospital, Harbin Medical University, Harbin, China

## Abstract

We sought to identify a serum miRNA expression profile to improve disease surveillance and to predict post-operative disease recurrence for stage II/III colorectal cancer (CRC) patients. Using the TaqMan Low-Density Array (TLDA), we performed an initial survey to analyze 749 miRNAs in the pooled serum of 20 paired pre- and post-operative CRC patients and 20 matched normal subjects. Using individual RT-qPCR verification in 175 stage II/III CRC patients, we identified that miR-145, miR-106a and miR-17-3p were significantly differentially expressed between pre- and post-operative CRC patients and between pre-operative CRC patients and normal controls (*P* < 0.0001). The area under the ROC curve (AUC) for the three-miRNA panel was 0.886 (95% CI 0.850–0.921) for discriminating between pre-operative CRC patients and normal subjects and 0.850 (95% CI 0.809–0.891) for discriminating between pre- and post-operative CRC patients. Furthermore, using the Kaplan-Meier method and Cox proportional hazards analysis, we found that miR-17-3p and miR-106a were powerful and independent prognostic indicators and that high levels of these miRNAs were associated with shorter disease-free survival (DFS) (*P* < 0.0001 for miR-17-3p and *P* = 0.001 for miR-106a). The present study reveals novel serum-miRNA-based biomarkers for monitoring tumor dynamics as well as for predicting disease recurrence in patients with stage II/III CRC.

Colorectal cancer (CRC) is currently the third most common malignancy and the second leading cause of cancer mortality worldwide, with more than 1.2 million new cases and 600,000 deaths annually^1^. Approximately 60% of CRC patients present with resectable disease when diagnosed, and for these patients, curative surgical resection followed by adjuvant chemotherapy is considered the standard treatment strategy. However, 20–30% of patients will ultimately develop recurrent disease, and the prognosis after recurrence is poor^2^,^3^. In addition, for patients with stage III CRC, a survival benefit from adjuvant chemotherapy has been firmly established in several large-scale trials^4^,^5^. However, a subgroup of these patients may have a low risk of recurrence because nearly 40% of the patients who were randomized to the observation arm in chemotherapy trials did not develop a recurrence^6^,^7^. For patients with stage II CRC, controversy exists as to the beneficial effect of post-operative adjuvant chemotherapy. Thus, the identification of biomarkers that predict which patients are more likely to develop recurrent disease is particularly important for stage II/III CRC patients. A blood-based test is particularly attractive because it is minimally invasive; however, currently, no clinically routine blood test is available. Therefore, the identification of novel biomarkers is evidently needed for the detection, monitoring and prediction of CRC to improve patient outcomes.

MicroRNAs (miRNAs) are a class of short (18–22 nt in length), evolutionarily conserved, non-protein-coding RNAs that play critical roles in diverse biological processes though negative post-transcriptional regulation^8^. Recent evidence has indicated that miRNAs can function as oncogenes or tumor suppressors by repressing cancer-related genes^9^. Alterations of miRNA expression have been observed in a variety of human tumors, including CRC, and we previously discovered that miRNAs are stably present in circulating blood at sufficient levels for use as blood-based biomarkers^10^. Consistent with these results, other studies found potential for serum-based miRNAs as biomarkers for cancer diagnosis^11^. In addition, previous studies have investigated the diagnostic potential of circulating miRNAs in CRC^12^,^13^,^14^,^15^,^16^. Recently, the role of miRNAs in predicting the response to 5-fluorouracil (5-FU)-based chemotherapy in CRC treatment has been explored. Unfortunately, studies determining how to monitor dynamic changes, assess prognosis and predict therapeutic outcomes in these patients are lacking.

In this study, we investigated the use of a serum miRNA profile as a prospective indicator to monitor disease dynamics and to predict disease-free survival by comparing the differential expression of serum miRNAs before and after curative resection for stage II/III colorectal cancer. We screened a serum miRNA-based expression profile using a high-throughput TaqMan Low-Density Array (TLDA), which was combined with a literature review, and validated the results in two independent patient groups using individual quantitative reverse-transcription PCR (RT-qPCR) assays.

## Results

### Patient characteristics

A total of 305 participants, including 175 CRC patients and 130 healthy controls, were enrolled in the present study. Table 1 shows the demographics and clinical features of the CRC patients and healthy controls in the two different cohorts. All 175 patients recruited for this study had undergone surgical resection with a histologically negative resection margin. The median follow-up was 36 months for the Tianjin cohort and 32 months for the Xiangya cohort, and 21% of the patients in the Tianjin cohort and 23% in the Xiangya cohort developed tumor relapse during the follow-up period. Of all 175 patients, 74 received adjuvant chemotherapy in our study: 43 of 85 in the Tianjin cohort and 31 of 90 in the Xiangya cohort. No significant differences were observed between CRC patients and normal controls in either the Tianjin cohort or the Xiangya cohort in the distribution of age (*P* = 0.49 and *P* = 0.56, respectively) or gender (*P* = 0.98 and *P* = 0.77, respectively).

### Screening of serum miRNAs for disease monitoring

**Figure 1:**
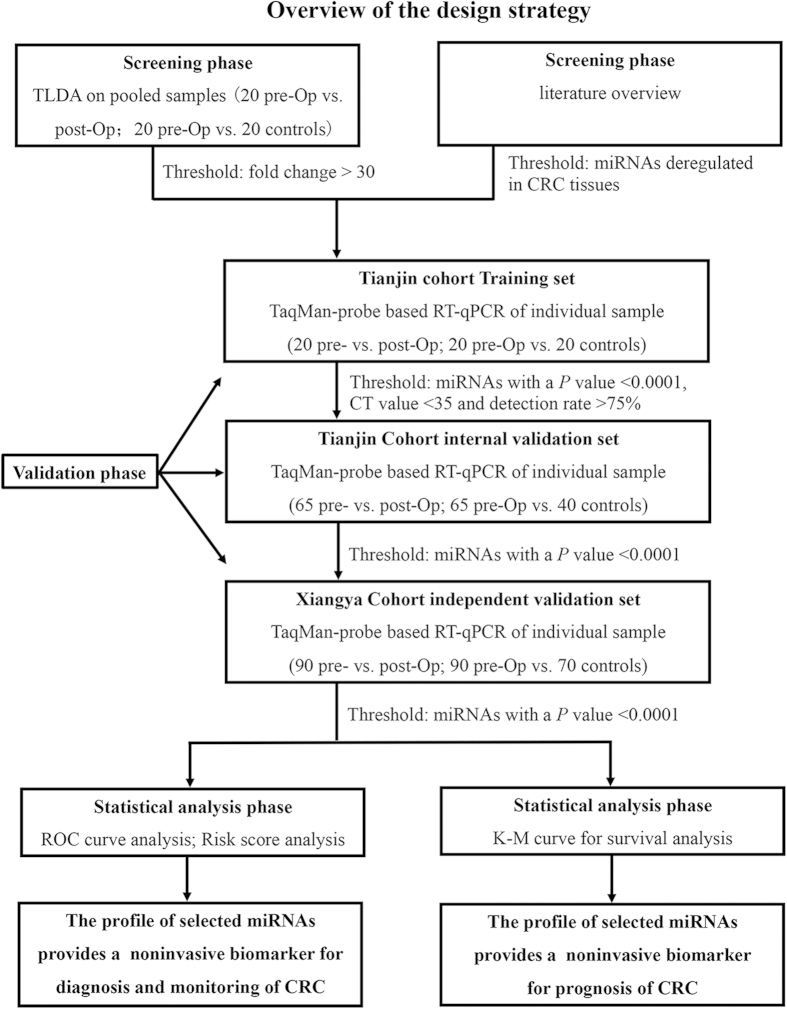
A flow chart of the study design strategy.

We designed a multiphase study to identify novel serum miRNAs as surrogate markers for detecting CRC and monitoring disease dynamics and predicting the prognosis of CRC patients (Fig. 1). To screen candidate serum miRNAs for CRC detection and monitoring, we combined the TLDA technique with a literature review. The miRNAs recruited in the present study and their reported relevance to CRC was seen in Supplemental Data Table S1. We employed the TLDA technique to screen the expression levels of 749 miRNAs in pooled serum samples from healthy controls and pre- and post-operative CRC patients (each pooled from 20 individuals). Compared with the healthy control group, serum from the pre-operative CRC group had 108 up-regulated miRNAs and 20 down-regulated miRNAs (fold change > 30) (See Supplemental Data Table S2). Moreover, 203 serum miRNAs were found to be differentially expressed between the pre-operative and post-operative groups, of which 183 were up-regulated and 20 were down-regulated (fold change > 30) (See Supplemental Data Table S3). We selected miRNAs that satisfied the following criteria for additional RT-qPCR validation: 1) fold change > 30 between the pre-operative CRC patients and the healthy controls; 2) fold change > 30 between pre- and post-operative CRC patients; and 3) decreased/increased serum levels of up-regulated/down-regulated miRNAs in the post-operative serum samples compared with the pre-operative samples that reached levels comparable to those of healthy controls. Using these criteria, 18 miRNAs were identified and chosen for further analysis (See Supplemental Data Table S4). In addition, we included six miRNAs (miR-17-3p, miR-21, miR-31, miR-92a, miR-143 and miR-145) that were previously reported to be dysregulated in CRC.

### Validation of candidate miRNAs by RT-qPCR

The differential expression of these 27 selected miRNAs was confirmed in two independent cohorts of 175 CRC patients and 130 healthy controls using RT-qPCR analysis. We first compared the miRNA profiles of 85 pairs of pre- and post-operative serum samples of CRC patients and 60 healthy controls in a training and internal validation set (including the individuals used to create the pool). At first, we validated the differential expression of these 27 selected miRNAs in 20 CRC patients and 20 healthy controls (samples that were used to create the pool) using RT-qPCR analysis in the training set. Differentially expressed miRNAs (*P* < 0.0001) were chosen only when they showed a parallel trend in the variation among the three groups in both the screening and validation phases. The expression levels of miR-15b, miR-17, miR-26b, miR-21, miR-31, miR-95, miR-139-3p, miR-143, miR-146b, miR-193a-3p, miR-199a-3p, miR-214, miR-216a, miR-337-5p and miR-382 were not significantly altered. The detection rates were <75% or the Ct values were higher than 35 in the quantitative RT-qPCR assay for miR-382, miR-412, miR-517b, miR-518b miR-572, miR-454, and miR-1233 (See Supplemental Table S5). The expression levels of miR-145, miR-17-3p and miR-106a were differentially expressed among three groups. Thus, we validated our results in a larger population in an internal validation set including 65 pairs of pre- and post-operative serum samples of CRC patients and 40 healthy controls. Ultimately, miR-17-3p and miR-106a were up-regulated in CRC patients and decreased after surgery, reaching levels comparable with those of healthy controls, whereas miR-145 was found to be down-regulated in CRC patients compared with those of normal subjects and reached levels comparable to those of normal subjects after surgery; thus, miR-17-3p, miR-106a and miR-145 were selected as candidates for further analyses.

**Figure 2:**
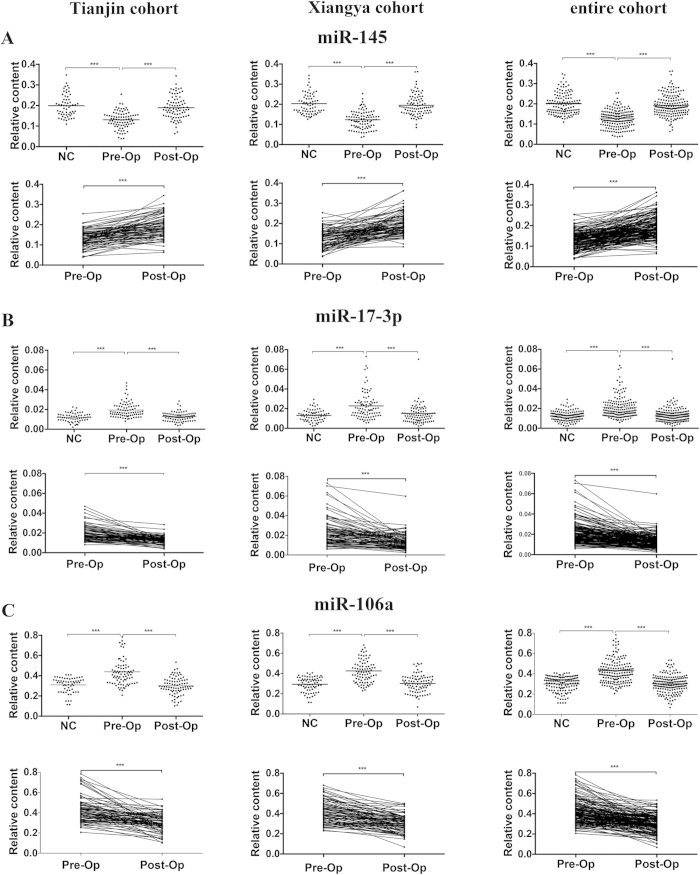
The differential expression of three serum miRNAs in pre-operative (pre-Op) and post-operative (post-Op) CRC serum samples and between CRC serum samples and control samples. Dot plots of the plasma levels of (**A**) miR-145, (**B**) miR-17-3p and (**C**) miR-106a in 175 CRC cases (paired pre- and post-operative samples) and 130 healthy controls (NC) (both from the Tianjin cohort and the Xiangya cohort) using RT-qPCR assays. The expression levels of the miRNAs were normalized to let-7d/g/i and calculated using the 2^−ΔΔCt^ method.

To confirm whether the miRNAs identified from the internal validation had similar alterations in different populations, we applied our internal validation set to the independent validation set of 90 patients from different healthcare centers. A set of 70 healthy subjects was recruited as the control group. We found similar results in this cohort: the expression levels of these three miRNAs were significantly altered in the post-operative samples compared with those in the pre-operative samples, reaching levels comparable with those of healthy controls (Fig. 2A–C). Together, these findings suggest that these miRNAs may be tumor-derived and have the potential to be novel indicators for monitoring disease dynamics.

To evaluate whether these selected miRNAs were consistently differentially expressed in colon and rectal cancers, we compared the levels of the three miRNAs in these two diseases. No significant difference was found between colon and rectal cancer (Supplemental Data Figure S1).

### Diagnostic accuracy of the candidate miRNAs

**Figure 3:**
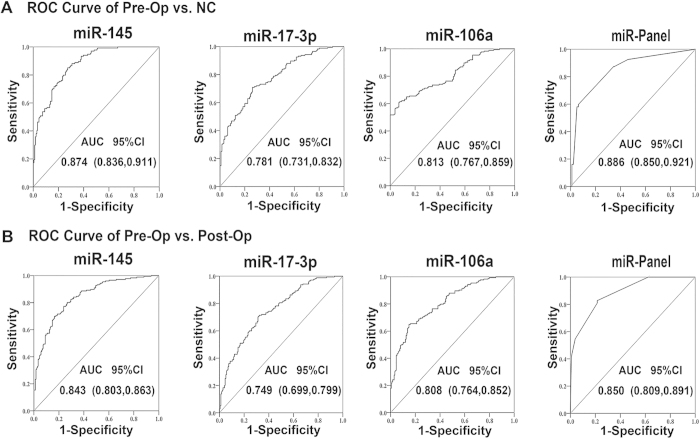
ROC curve analysis for the discrimination between pre-operative (pre-Op) and post-operative (post-Op) CRC serum samples and between CRC serum samples and control samples based on the three-serum miRNA profile. (**A**) ROC curve for the ability of miR-145, miR-17-3p, miR-106a and the miR panel to separate the 175 pre-operative (pre-Op) from post-operative (post-Op) patients. (**B**) ROC curve for the ability of each miRNA and the miR panel to discern 175 CRC cases from 130 healthy controls (NC).

A ROC curve analysis was used to evaluate the diagnostic accuracy of the selected serum miRNAs between CRC patients and normal subjects. Compared with normal subjects, the AUCs for miR-145, miR-106a and miR-17-3p were 0.874, 0.808 and 0.781, respectively. Furthermore, the AUC for the combination of the three miRNAs was 0.886 (95% CI 0.850–0.921) (Fig. 3A). With an optimal cutoff value of 1.613, at which the sum of the sensitivity and specificity was maximal, the specificity was 0.828 and the sensitivity was 0.785 for CRC. Together, these results indicate that the identified miRNAs, alone or in combination, can discriminate between CRC cases and normal samples with high accuracy. Concurrently, using ROC curve analysis, we assessed the discriminating power of the candidate miRNAs to distinguish pre- from post-operative CRC patients. Our results showed a relatively high accuracy for each miRNA and for the combined three-miRNA panel, with AUCs of 0.843, 0.813, 0.749 and 0.850 for miR-145, miR-106a, miR-17-3p and the combined miRNA panel, respectively (Fig. 3B). Together, these results suggest that the three miRNAs can discriminate CRC patients with relatively high accuracy and could be powerful indictors for CRC monitoring.

### Prognostic indicators for disease relapse

**Figure 4:**
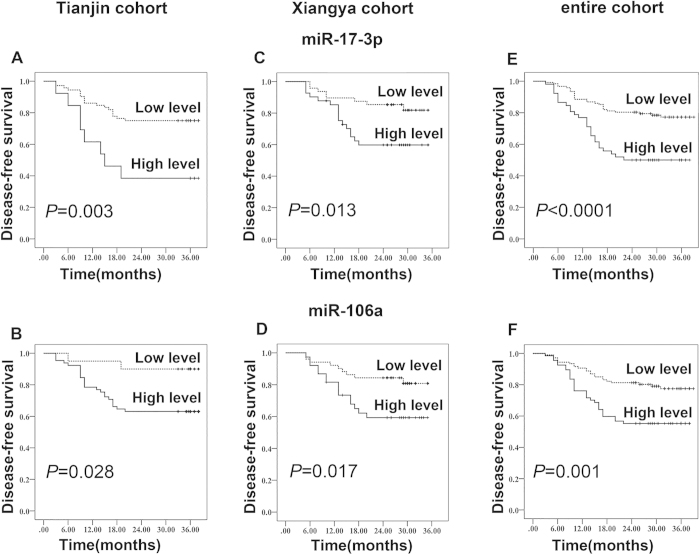
A Kaplan–Meier curve estimates the association of miRNAs and the survival of patients with stage II/III colorectal cancer. (**A**–**C**) The miR-17-3p high-level group (greater than the cutoff value) compared with the low-level group (less than the cutoff value) in both cohorts. (**D**–**E**) The miR-106a high-level group compared with the low-level group in both cohorts.

We then performed an analysis to identify whether the three selected miRNAs were associated with cancer recurrence. We analyzed the expression level of each individual miRNA for an association with poor prognosis in both the Tianjin cohort and the Xiangya cohort using Kaplan–Meier DFS curves for all of the recruited CRC patients. The median follow-up period was 36 months for the Tianjin cohort and 32 months for the Xiangya cohort. We categorized these patients into patients at high risk of disease recurrence (high-risk group) and patients at low risk of disease recurrence (low-risk group) based on the expression levels of each miRNA. When we assessed the distribution of the expression levels of each miRNA and survival status, we found similar results for the prognostic values in both the Tianjin cohort and the Xiangya cohort. In the Tianjin cohort, patients with lower levels of miR-17-3p and miR-106a generally had better survival than patients with higher levels (log-rank test: *P* = 0.005 and *P* = 0.028, respectively) (Fig. 4A,B). In the Xiangya cohort, we also found consistent correlations between the expression level of each miRNA and survival status (log-rank test: *P* = 0.013 for miR-17-3p and *P* = 0.017 for miR-106a) (Fig. 4C,D). Similar results were found in all 175 patients (log-rank test: *P* < 0.0001 for miR-17-3p and *P* = 0.001 for miR-106a) (Fig. 4E,F). Subsequently, we performed univariate and multivariate Cox proportional hazards regression analyses to determine the influence of serum miRNA levels and clinicopathological characteristics on patient survival. In the univariate analysis for the Tianjin test cohort, we found that high expression of miR-17-3p and miR-106a as well as TNM stage was significantly correlated with DFS (*P* = 0.002, *P* = 0.03 and *P* = 0.001, respectively). Similar results were also observed in an independent validation of miR-17-3p and miR-106a levels as well as TNM stage for the Xiangya cohort (*P* = 0.009, *P* = 0.023 and *P* = 0.007, respectively). After multivariable adjustment by clinicopathological variables, miR-17-3p, miR-106a and the TNM stage remained independent risk factors in the entire cohort of all 175 patients (HR 2.24, 95% CI 1.28-3.92, *P* = 0.035; HR 3.02, 95% CI 1.36-6.73, *P* = 0.007; and HR 4.13, 95% CI 1.66-10.2, *P* = 0.002, respectively). We also found similar results in the combined training and internal validation set (*P* = 0.012, *P* = 0.013 and *P*  = 0.001, respectively) and in the independent validation set (*P* = 0.011, *P* = 0.01 and *P*  = 0.002, respectively) (Table 2). These data indicate that miR-17-3p and miR-106a may be prognostic biomarkers for CRC patients with localized disease who underwent surgery.

### Prognostic indicators for therapeutic outcome

**Figure 5:**
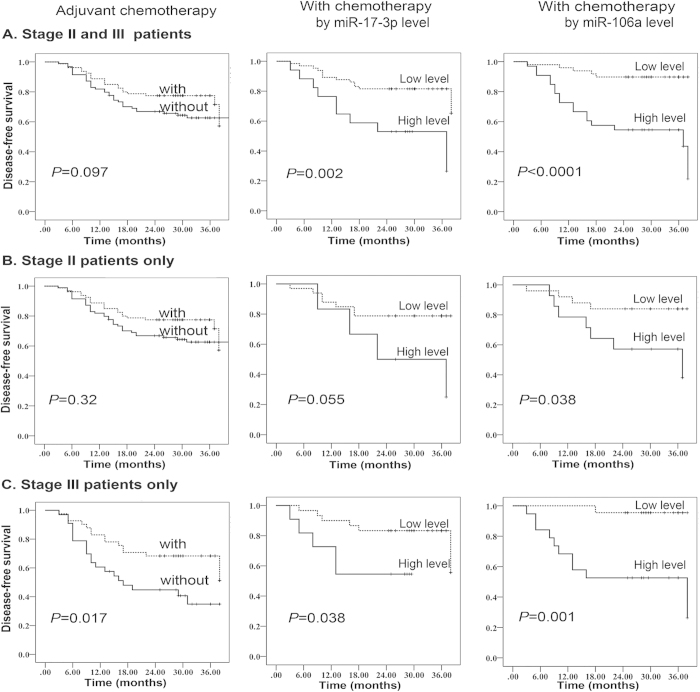
A Kaplan–Meier curve estimates the association of miRNAs and the survival of patients with stage II/III colorectal cancer who received adjuvant chemotherapy. (**A**) Association between the expression of miR-17-3p and miR-106a and disease-free survival (DFS) in patients with stage II/III CRC who had received chemotherapy. (**B**) Associations between the expression of miR-17-3p and miR-106a and DFS in patients with stage II CRC who had received chemotherapy. (**C**) Association between the expression of miR-17-3p and miR-106a and DFS among patients with stage III CRC who had received chemotherapy.

We analyzed the associations between miRNA expression and therapeutic outcomes in stage II and III cancer patients treated with adjuvant chemotherapy. Receipt of adjuvant chemotherapy was beneficial for patients with either stage II or stage III cancer, although this benefit was only significant for stage III patients (*P* = 0.017). For individuals who received adjuvant therapy, high expression of miR-17-3p or miR-106a predicted a more rapid tumor recurrence (*P* = 0.002 and *P* < 0.0001, respectively), providing preliminary support that high levels of miR-17-3p and miR-106a are associated with poor therapeutic outcomes (Fig. 5A). Additionally, because the benefit from adjuvant chemotherapy was not consistent for patients with different disease stages, we then separately evaluated the association of miRNA levels and therapeutic outcomes in patients with stages II and III. Of the 39 patients with stage II CRC who had received adjuvant chemotherapy, high levels of miR-17-3p and miR-106a were associated with poor therapeutic outcomes (*P* = 0.055 and *P* = 0.038, respectively) (Fig. 5B). For 41 patients with stage III CRC who had received adjuvant chemotherapy, high levels of miR-17-3p and miR-106a were also associated with shorter DFS (*P* = 0.038 and *P* = 0.001, respectively) (Fig. 5C). These results show that miR-17-3p and miR-106a were associated with poor therapeutic outcomes in CRC patients with localized disease who had already undergone curative surgical resection and received adjuvant chemotherapy.

## Discussion

Previous studies have identified multiple circulating miRNAs that are dysregulated in CRC patients compared with normal controls. However, few studies have evaluated the dynamic changes in miRNAs before and after curative surgical resection of a primary tumor^12^,^14^,^17^,^18^,^19^. Of these studies, only two, with sample sizes of 10 and 20 CRC patients, have assessed the differential expression of miRNAs before and after surgical cancer resection^12^,^17^. In the present study, we compared the differential expression levels of a circulating miRNA profile between pre- and post-operative blood samples of CRC patients and matched healthy individuals in two large-scale, independent cohorts. We performed the largest study to date that analyzed the distinctive expression of circulating miRNA profiles in paired pre- and post-operative blood samples from patients with colorectal cancer using two independent cohorts. What’s more, the expression levels of these miRNAs were consistently altered both in colon and rectal cancers and no difference between them. In present study, we found a novel panel of circulating miRNAs that remarkably distinguished CRC patients before and after surgery, indicating it may reflect the tumor burden. Using the three miRNA-based panel may help to monitor the effect of surgical resection with high accuracy in clinical practice. Our findings suggest that these miRNAs could potentially be used to monitor disease dynamics, such as estimating the completeness of surgical resection and monitoring tumor recurrence in CRC with localized disease. Future studies may further evaluate its potential use in post-operative surveillance, which may provide an opportunity for the early detection of recurrent disease.

In addition, although many studies have evaluated the diagnostic potential of circulating miRNAs in CRC, the relation of circulating miRNA deregulation with disease outcome or response to chemotherapy in colorectal cancer patients has been explored in only a few studies^20^,^21^. Our studies also demonstrated the potential value of serum miRNAs for predicting tumor relapse as well as therapeutic outcome in CRC patients with localized disease. We observed that high levels of circulating miR-17-3p and miR-106a were associated with poor survival, and this association was independent of other clinical factors, indicating the potential of these miRNAs as noninvasive prognostic biomarkers for CRC. In addition, for patients who undergo adjuvant chemotherapy, high levels of miR-17-3p and miR-106a predicted a poor therapeutic outcome. This association may help predict the benefits of therapy in individuals whose expression status is known and identify patients who are candidates for more intensive initial therapies. If high expression is causal to a poor therapeutic outcome, antagomirs that target these two miRNAs may prove to have therapeutic benefits in patients with tumors with high expression levels.

Appropriate normalization using stably expressed reference genes is important and critical for the accurate quantification of RNA levels when using RT-qPCR. A common problem in research on circulating miRNAs is that no consensus endogenous controls have been established to date. The identified studies used several different genes as their endogenous controls, such as RNU6B, RNU44, RNU48 and miR-16^22^,^23^. However, few reports detailed a robust identification and validation strategy for suitable reference genes for normalization. Our laboratory has clearly demonstrated that a combination of let-7d, let-7g and let-7i can be a suitable reference for the normalization of serum miRNAs and that this combination is statistically superior to the commonly used reference genes U6, RNU44, RNU48 and miR-16^24^. Thus, we used let-7d/g/i as an endogenous control for the normalization of serum miRNA levels to guarantee the reliability of our results.

The biological relevance and the expression pattern in CRC tumor tissue of the selected miRNAs identified in our study have been widely investigated in previous studies. MiR-145 has been reported to possess anti-tumorigenic activity and be involved in a variety of cancer-related biological processes during the tumorigenesis and progression of various cancers, including colorectal cancer; this miRNA has been shown to exhibit reduced accumulation in colorectal tumor tissue compared with adjacent non-tumor tissue^25^,^26^,^27^. In addition, previous studies have investigated several miR-145 targets that possess vital roles in colorectal cancer^28^,^29^,^30^,^31^. We speculated that the dynamic change of circulating miR-145 may help monitoring disease dynamics in clinical diagnosis; however, we could not make hypothesis that this alteration was due to the reduced or increased secrete by tumor cells. We envision that the exact molecular mechanism for this alteration needs further exploration in future studies. Previous studies have found that miR-106a is up-regulated in CRC tumor tissue and that deregulation of miR-106a was associated with survival in CRC patients^32^,^33^,^34^. MiR-17-3p is a member of the miR-17-92a cluster, which has been reported to play oncogenic roles by promoting cell proliferation, suppressing apoptosis of cancer cells, and inducing tumor angiogenesis. The overexpression of this cluster has been observed in several types of cancer, including CRC^35^,^36^,^37^. These data indicate that the overexpression of these two miRNAs (miR-17-3p and miR-106a) in serum may be tumor-derived, which strengthens their clinical application as noninvasive biomarkers that are specific for CRC. Further studies should be performed to reveal the underlying mechanism for the deregulation of circulating miRNAs.

The present work has some limitations. Previous studies have found rectal cancer-specific miRNAs in rectal cancer tissues compared with normal tissues^38^. However, the expression levels of these three miRNAs in our study were not significantly different between colon and rectal cancer, which indicates that these three miRNAs may reflect the common biological process in colon and rectal cancer and could be biomarkers for these two cancers. Future studies should be designed to find the colon or rectal cancer-specific cancer. Additionally, the present work failed to evaluate the miRNA expression profiles in different ethnic population, which limits the potential use of these three miRNAs in different racial groups. Nevertheless, our group recently has evaluated the levels of serum miRNAs in different racial populations and found that there is no significant difference after normalizing by the endogenous control let-7d/g/i. Future studies should include a validation to evaluate the expression levels of these miRNAs in a similar large cohort in different ethnic groups.

In conclusion, we found a novel panel of distinctive circulating miRNAs by comparing paired pre- and post-operative blood samples from stage II/ III CRC patients. High expression levels of circulating miR-17-3p and miR-106a were correlated with poor survival and therapeutic outcome. These results indicate that circulating miRNAs have the potential to be used for monitoring tumor dynamics and as predictive biomarkers for prognosis and therapeutic outcomes in CRC patients who received chemotherapy.

## Materials and Methods

### Study design, patients and control subjects

We used paired serum samples of 175 stage II/III CRC patients before and 7-14 days after curative resection. In the initial biomarker screening stage, we subjected pooled serum samples from 20 CRC cases both before and after surgery (Tianjin Medical University Cancer Institute and Hospital, Tianjin) and from 20 matched controls (Jinling Hospital, Nanjing) to identify miRNAs whose expression was altered in CRC. For the training and internal validation set, 85 patients were recruited from Tianjin Medical University Cancer Institute and Hospital, Tianjin, China, between May 4, 2011, and Aug 20, 2011. In addition, 60 matched, disease-free individuals were recruited as healthy controls. In the external validation set, we included another 90 patients from the Affiliated Tumor Hospital of Xiangya Medical School of Central South University and from the Third Xiangya Hospital of Central South University, Changsha, between Dec 20, 2011, and May 5, 2012. Another 70 healthy individuals (Jinling Hospital) were enrolled as controls.

Patients were excluded if they had any tumor type other than adenocarcinoma or mucinous adenocarcinoma, acute infections, or pre-operative chemotherapy or radiotherapy. Tumor stage was determined based on surgical findings, and tumors were staged according to the tumor-node-metastasis (TNM) system classification of the American Joint Committee on Cancer^39^. Detailed clinical features for each patient, including age, gender, clinical stage, tumor size, tumor location, survival time and receipt of adjuvant chemotherapy, were available. The final date of follow-up was April 30, 2014, for the Tianjin cohort and May 30, 2014, for the Xiangya cohort. Disease-free survival (DFS) was defined as the time from the date of surgery to the date of confirmed tumor relapse or death from tumor relapse. DFS was censored at the date of death from other causes or at the date of the final follow-up visit for disease-free patients. Information on the administration of adjuvant chemotherapy was available for all patients in both cohorts. Chemotherapy regimens were primarily fluorouracil-based and with or without oxaliplatin. All 130 healthy individuals in this study were recruited from a large pool of individuals seeking a routine health check-up at the Healthy Physical Examination Center of Jinling Hospital. Subjects with no evidence of disease were enrolled as cancer-free controls. All subjects provided written informed consent to participate in the study. The methods in this study were carried out in accordance with the approved guidelines by Tianjin Medical University Cancer Institute and Hospital, Affiliated Tumor Hospital of Central South University, the Third Xiangya Hospital of Central South University and Jinling Hospital and all experimental protocols were approved by the ethics committees of the four hospitals. This study was approved by the ethics committees of each of the participating institutions.

### Lirerature overview

To identify all relevant literature, we searched PubMed for CRC miRNA expression profiling studies published between 2000 and 2012.We carried out a systematic review to determine the differentially expressed up-regulated and down-regulated miRNAs that were consistently reported in independent miRNA expression profiling studies in CRC patients and selected most up-regulated and down-regulated miRNAs (at least four studies). Three miRNAs (miR-106a, miR-21, miR-31) were the most up-regulated and four miRNAs (miR-30a-3p, miR-145, miR-125a and miR-133a) were the most down-regulated in colon tumor tissue. In addition, we further select those that play vital roles in colon cancer. Finally, miR-145, miR-143, miR-21 and miR-31 were selected in our studies. The miRNAs recruited in the present study and their reported relevance to CRC was seen in Supplemental Data Table S1. Besides, previous study has shown that serum miR-17-3p and miR-92a were differential expressed before and after surgical resection in CRC patients but with only small samples^12^. We also recruited these two miRNAs for validation in a large-scale population.

### Blood collection and rna isolation

The methods used for sample processing are given in the Supplemental Data file in the online Data Supplement. All the analyses for miRNAs were performed in a central laboratory (Jiangsu Engineering Research Center for microRNA Biology and Biotechnology, State Key Laboratory of Pharmaceutical Biotechnology, School of Life Sciences, Nanjing University, Nanjing, China). For the TLDA analysis, equal volumes of serum before and after surgery from 20 CRC patients and 20 controls (500 μL each) were pooled (10 mL each). TRIzol reagent (Invitrogen, Carlsbad, CA) was used to extract total RNA from each pool of serum samples as previously described^10^. The aqueous phase was subjected to 3 steps of acid phenol/chloroform purification to eliminate protein residue before isopropyl alcohol precipitation. The resulting RNA pellet was dissolved in 20 μL of RNase-free water and stored at −80 °C until further analysis.

For the RT-qPCR assay, total RNA was extracted from 100 μL of serum with a 1-step phenol/chloroform purification protocol as previously described^40^. In brief, 100 μL of serum was mixed with 200 μL of acid phenol, 200 μL of chloroform, and 300 μL of RNase-free water. The mixture was vigorously vortex-mixed and centrifuged at room temperature for 15 min. After phase separation, the aqueous layer was mixed with 1.5 volumes of isopropyl alcohol and 0.1 volumes of 3 μmol/L sodium acetate (pH 5.3), and the solution was stored at −20 °C for 1 h. The RNA pellet was collected by centrifugation at 16,000 g for 20 min at 4 °C and then washed once with 750 μL/L ethanol and dried for 10 min at room temperature. Finally, the pellet was dissolved in 20 μL of RNase-free water and stored at −80 °C until further analysis.

### Analysis of miRNAs by tlda

TLDA analysis was performed as previously described^41^. For more details, see the Supplemental Data file in the online Data Supplement.

### Analysis of serum miRNAs by RT-qPCR

A TaqMan probe–based RT-qPCR assay was performed according to the manufacturer’s instructions (7500 Sequence Detection System; Applied Biosystems), with minor modifications (see Methods in the Supplemental Data file in the online Data Supplement). A combination of let-7d, let-7g and let-7i (let-7d/g/i) showed high stability across normal controls and patients with disease and was statistically superior to the most commonly used reference genes for the quantification of serum miRNAs^24^. Therefore, let-7d/g/i was used as an endogenous control to normalize the data from the experimental RT-qPCR. The relative levels of miRNA were normalized to let-7d/g/i and were calculated using the equation 2^−ΔΔCt^. ΔCt was calculated by subtracting the Ct values of let-7d/g/i from the average Ct values of the miRNAs of interest. ΔΔCt was then calculated by subtracting the ΔCt of the controls from the ΔCt of the cases.

### Data analysis

All statistical analyses were performed using the Statistical Analysis System software SPSS (version 20.0, SAS Institute). Data are presented as the means ± SEMs for miRNAs or as the means ± SDs for other variables. We compared two groups using Student’s *t*-test for continuous variables and the two-sided χ^2^ test for categorical variables. Statistical significance was set at 0.05. We constructed receiver-operating-characteristic (ROC) curves and calculated the area under the ROC curves (AUC) to evaluate the predictive power of candidate miRNAs for CRC. We performed risk score analysis to evaluate the associations between the miRNA expression level and CRC (see Methods in the Supplemental Data file in the online Data Supplement). To evaluate the association between the expression levels of selected miRNAs and patient survival, we categorized these patients into patients at high risk of disease recurrence (high-risk group) and patients at low risk of disease recurrence (low-risk group) based on the expression levels of each miRNA. Expression levels of these three miRNAs were stratified by the median value and the association between survival outcome and miRNA levels was determined using the Kaplan-Meier method. We also calculated the level of statistical significance for each miRNA, based on univariate and multivariate Cox proportional hazard regression models. Statistical significance was calculated using the log-rank test. Stepwise univariate and multivariate Cox regressions were performed to analyze factors related to DFS.

## Additional Information

**How to cite this article**: Li, J. *et al.* Serum miRNA expression profile as a prognostic biomarker of stage II/III colorectal adenocarcinoma. *Sci. Rep.* **5**, 12921; doi: 10.1038/srep12921 (2015).

## Supplementary Material

Supplemental Data

## Figures and Tables

**Table 1:** Demographic and clinical features of CRC patients and healthy subjects^a^.

Variables	Internal Validation set (n = 85)	Independent Validation set (n = 90)	Healthy Subjects (n = 130)
Age (years)			
Mean (SD)	58.7(12.3)	56.6(10.7)	54.1(7.5)
≥65, No.	26	28	51
<65,No.	60	62	79
Sex, No.			
Male	53	60	70
Female	32	30	50
Follow-up time, months			
Median	36.0	32.0	
Range	33.0–38.1	27.5–35.0	
Recurrence rates			
1 year	13/85	10/89	
3 year	19/85	23/89	
Histological grade, No.			
Middle-Low	76	74	
High	9	16	
Tumor location, No.			
Colon	37	37	
Rectum	49	53	
TNM stage, No.			
II	52	49	
III	34	41	
Adjuvant chemotherapy			
Received	37	43	
Did not received	48	42	

^a^Data are presented as Mean ± SD.

**Table 2:** Univariate and multivariate analyses of parameters associated with disease-free survival (DFS) of all CRC patients^a^.

Characteristic	Univariate Analysis		Multivariate Analysis	
	HR^1^ (95% CI^2^)	*P-*value	HR (95% CI)	*P-*value^b^
Tianjin Training and Internal Validation Cohort				
Age (≥65/<65)	1.11(0.48-2.55)	0.81	1.39(0.54-3.56)	0.49
Gender (male/female)	0.77 (0.36-1.68)	0.518	0.80 (0.33-1.92)	0.62
Stage (III/II)	3.54 (1.75-7.81)	0.001	4.54 (1.93-10.6)	0.001
Histology (high/low)	3.99 (0.88-5.88)	0.072	3.56 (1.27-10.1)	0.017
Location (colon/rectum)	1.06 (0.49-2.30)	0.873	1.11 (0.48-2.54)	0.79
miR-145 (low/high)	0.68 (0.30-1.53)	0.35	0.67 (0.27-1.68)	0.39
miR-17-3p (low/high)	3.72 (1.61-8.60)	0.002	3.74 (1.34-10.4)	0.012
miR-106a (low/high)	4.31 (1.02,18.27)	0.03	3.34 (1.29-8.62)	0.013
Xiangya Independent Validation Cohort				
Age (≥65/<65)	0.72 (0.25-2.11)	0.551	1.36 (0.42-1.91)	0.61
Gender (male/female)	0.64 (0.25-1.60)	0.336	2.17 (0.79-5.95)	0.13
Stage (III/II)	3.36 (1.39-8.10)	0.007	5.12 (1.83-14.2)	0.002
Histology (high/low)	0.86 (0.39-1.92)	0.718	1.24 (0.50-3.05)	0.64
Location (colon/rectum)	1.13 (0.50-2.55)	0.764	2.39 (0.90-6.39)	0.08
miR-145 (low/high)	1.42 (0.64-3.17)	0.39	0.77 (0.29-1.98)	0.58
miR-17-3p (low/high)	3.09 (1.33-7.24)	0.009	3.74 (1.34-10.4)	0.011
miR-106a (low/high)	2.61 (1.14-5.98)	0.023	3.34 (1.28-8.63)	0.01
All recruited CRC patients				
Age (≥65/<65)	1.02 (0.99-1.04)	0.142	1.05 (0.99-1.11)	0.09
Gender (male/female)	0.64 (0.25-1.60)	0.336	1.85 (0.48-9.43)	0.54
Stage (III/II)	1.68 (1.16-2.44)	0.006	4.13 (1.66-10.2)	0.002
Histology (high/low)	4.29 (0.95,19.8)	0.059	1.62 (0.71-3.12)	0.098
Location (colon/rectum)	0.87 (0.44-2.01)	0.874	1.47 (0.62-3.44)	0.38
miR-145 (low/high)	0.78 (0.46-1.35)	0.382	0.61 (0.26-1.37)	0.228
miR-17-3p (low/high)	2.72 (1.58-4.69)	<0.0001	2.24 (1.28-3.92)	0.035
miR-106a (low/high)	2.81 (1.64-4.80)	<0.0001	3.02 (1.36-6.73)	0.007

^a^Data are normalized to let-7d/g/i and presented as the mean ± SEM.

^b^Mann-Whitney unpaired test for rank sum.
