# Supplementary material for: Serum miRNA expression profile as a prognostic biomarker of stage II/III colorectal adenocarcinoma
Source: Sci Rep. 2015 Aug 7;5:12921. doi: 10.1038/srep12921 (PMC4528229; doi:10.1038/srep12921)
Supplement: Supplemental Data [file srep12921-s1.pdf]

Serum miRNA expression profile as a prognostic biomarker of stage II/III colorectal adenocarcinoma

Jialu Li [a,b,†], Yang Liu [c,†], Cheng Wang [d,†], Ting Deng [a,†], Hongwei Liang [b], Yifei Wang[e], Dingzhi Huang [a], Qian Fan [a,b], Xia Wang [a], Tao Ning [a], Rui Liu [a], Chen-Yu Zhang [b,*], Ke Zen [b,*], Xi Chen [b,*] and Yi Ba [a,*]

[a] Tianjin Medical University Cancer Institute and Hospital, Key Laboratory of Cancer Prevention and Therapy, Huanhuxi Rd., Tiyuanbei, Tianjin 300060, China.

[b] Jiangsu Engineering Research Center for microRNA Biology and Biotechnology, State Key Laboratory of Pharmaceutical Biotechnology，School of Life Sciences, Nanjing University, 22 Hankou Rd., Nanjing 210093, Nanjing, China.

[c] Department of Pathology, The Third Xiangya Hospital, Central South University, Changsha, Hunan, China.

[d] Department of Clinical Laboratory, Jinling Hospital, Clinical School of Medical College, Nanjing University, Nanjing, China.

[e] Department of Neurology, Second Affiliated Hospital, Harbin Medical University, Harbin, China.

[†]These authors contributed equally to this work.

Correspondence to: Yi Ba. E-mail address: bayi@tjmuch.com. Xi Chen, Ke Zen and Chen-Yu Zhang. E-mail address: xichen@nju.edu.cn. kzen@nju.edu.cn. and

cyzhang@nju.edu.cn.

Supplemental Data Methods

Sample processing

Five mL of venous blood was collected from each donor and placed in a separating gel vacuum tube. To harvest the cell-free serum, each blood sample was immediately centrifuged at 3,000 rpm for 10 min, followed by a second centrifugation at 12,000 rpm for 5 min. The serum supernatant was recovered and stored at -80°C until further analysis.

TDLA technology

TDLA technology was performed as follow descriptions. Briefly, 3 μL total RNA were added to 4.5 μL of the RT reaction mix (Megaplex RT Primers 10X, dNTPs with dTTP 100mmol, MultiScribe Reverse Transcriptase 50U/μL, 10X RT Buffer, MgCl2 25 mmol, RNase Inhibitor 20 U/μL and nuclease-free water). After incubation on ice for 5 min, reverse transcription was performed using a thermal cycler (UNO-ThermobloCk, Biometra, Göttingen, Germany). MicroRNA profiling of 739 different human miRNAs was then performed using the TaqMan Low Density Array (TaqMan Array Human MicroRNA A+B Cards Set v3.0). In order to generate enough miRNA cDNA templates for the following real-time RCR, a pre-amplification was performed after the reverse transcription. All steps were performed using a 7900 HT Fast Real-Time PCR System (Applied Biosystems, Foster City, CA) and all reactions were performed following the protocols of the manufacturer. The expression data for the miRNA were presented as threshold cycle (CT) values.

Quantification of miRNAs by probe-based RT-qPCR

A TaqMan probe-based RT-qPCR assay was performed according to the manufacturer's instructions (Applied Biosystems) with a minor modification. The resulting Ct values were determined using fixed threshold settings. Briefly, the reverse transcription reaction was carried out in 10 μL mixture containing 2 μL of extract RNA from serum, 1 μL of 10 mmol/L dNTPs, 0.5 μL of AMV reverse transcriptase (TaKaRa), 1 μL of a stem-loop RT primer (Applied Biosystems), 2 μL of 5 × reverse transcription buffer and 3.5 μL of diethylpyroCarbonate (DEPC)-treated water. For synthesis of cDNA, the reaction mixtures were incubated at 16 ^{o}C for 30 min, at 42 ^{o}C for 30 min, at 85 ^{o}C for 5 min, and then held at 4 ^{o}C. Real-time PCR was then performed (1 cycle of 95 ^{o}C for 5 min, and 40 cycles of 95 ^{o}C for 15 sec and 60 ^{o}C for 1 min) with an Applied Biosystems 7500 Sequence Detection System. The reaction was performed with a final volume of 20 μL containing 1 μL of cDNA, 0.3 μL of Taq, 0.33 μL of hydrolysis probe (Applied Biosystems), 1.2 μL of 25 mmol/L $MgCl_2$, 0.4 μL of 10 mmol/L dNTPs, 2 μL of 10 × PCR buffer, and 14.77 μL of DEPC water. All reactions, including controls no template RNA, were performed in triplicate.

Risk score analysis

Risk score analysis was performed to evaluate the associations between the expression levels of the serum miRNAs and CRC. The risk score of each miRNA, denoted as s, was set to 1 if the expression level was greater than the upper 95% reference interval for the corresponding miRNA level in controls and to 0 otherwise. A risk score function (RSF) to predict CRC was defined according to a linear

combination of the expression level for each miRNA. For example, the RSF for sample i using information from six miRNAs was: rsfi=∑6j-1Wj.sij. In the above equation, sij is the risk score for miRNA j on sample i, and Wj is the weight of the risk score of miRNA j. To determine the Ws, six univariate logistic regression models were fitted using the disease status with each of the risk scores. The regression coefficient of each risk score was used as the weight to indicate the contribution of each miRNA to the RSF. Samples were ranked according to their RSF and then divided into a high-risk group, representing the predicted CRC cases, and a low-risk group, representing the predicted control individuals. We then used frequency tables and ROC curves to evaluate the diagnostic effects of the profiling and to find the appropriate cutoff point.

Supplemental Data Table S1

The miRNAs recruited in the present study and their reported relevance to CRC.

MiRNA	Function	Differential expression	Prognosis
miR-21	Promoting the proliferation invasion and reducing apoptosis of colon cancer (Asangani IA et al., 2008; Liu M et al., 2011; Bakirtzi K et al., 2011; Zhang J et al., 2012; Faltejskova P et al., 2012; Vicinus B et al., 2012; Yu Y et al., 2012;)	Up-regulation in colon tumor tissue (Slaby O et al., 2007; Schetter et al., 2008; Wang P et al., 2009; Schetter AJ et al., 2009; Baffa R et al., 2009; Chang KH et al., 2010; Chang KH et al., 2011; Schee K et al., 2012; Kanaan et al., 2012； Faltejskova P et al., 2012	High miR-21 expression associated with poor survival and poor therapeutic outcome (Schetter AJ et al., 2009) Expression levels was higher associated with shorter disease-free survival (Nielsen BS et al., 2011) High expression level of miR-21 associated with poor survival (Kanaan et al., 2012)
miR-31	Promoting proliferation and tumor cell survival (Kallioniemi O et al., 2012; Chen T)	Up-regulation in colon tumor tissue (Bandrés E et al.,2006; Slaby O et al., 2007; Wang CJ et al., 2009； Motoyama K et al., 2009; Chang KH et al., 2011; Schee K et al., 2012	
miR-143	Inbitory activity of proliferation in colon cancer cell (Akao Y et al., 2010; Borralho PM et al., 2011; Zhang Y et al., 2012; Bauer KM1 et al., 2012; Gregersen LH et al.; 2012 Li X et al., 2012)	Down-regulation in colon tumor tissue (Slaby O et al., 2007; Wang CJ et al., 2009; Chen X et al., 2009; Motoyama K et al., 2009; Chang KH et al., 2011	
miR145	Antitumor effect in human colon cells (Akao Y et al., 2010; Zhang J et al., 2011; Wang Z et al., 2012; Xu Q et al., 2012)	Down-regulation in colon tumor tissue (Michael MZ et al., 2003; Bandrés E et al., 2006; Slaby O et al., 2007; Schepeler T et al., 2008; Wang CJ et al., 2009; Motoyama K et al., 2009)	

Supplemental Data Table S2

Differentially expressed miRNAs in pre-operative CRC serum samples compared to healthy control serum samples determined by TLDA.

miRNA	Ct of healthy control	Ct of pre-operative CRC	-ΔCt (pre-operative - control)
hsa-miR-337-5p	40.00	15.74	24.26
hsa-miR-216a	40.00	18.86	21.14
hsa-miR-193a-3p	40.00	19.79	20.21
hsa-miR-504	40.00	20.57	19.43
hsa-miR-17	40.00	21.95	18.05
hsa-miR-106a	40.00	21.96	18.04
hsa-miR-382	40.00	22.79	17.21
hsa-miR-1233	40.00	22.95	17.05
hsa-miR-21	40.00	22.97	17.03
hsa-miR-380-3p	40.00	22.98	17.02
hsa-miR-654-3p	40.00	23.62	16.38
hsa-miR-208b	40.00	23.73	16.27
hsa-miR-409-5p	40.00	24.93	15.07
hsa-miR-122	40.00	24.96	15.04
hsa-miR-331-5p	40.00	24.96	15.04
hsa-miR-1260	40.00	25.04	14.96
hsa-miR-515-5p	40.00	25.72	14.28
hsa-miR-142-5p	40.00	25.95	14.05
hsa-miR-214	40.00	26.00	14.00
hsa-miR-486	40.00	26.01	13.99
hsa-miR-1305	40.00	26.02	13.98
hsa-miR-139-3p	40.00	26.03	13.97
hsa-miR-672	40.00	26.29	13.71
hsa-miR-412	40.00	26.81	13.19
hsa-miR-365	40.00	27.01	12.99
hsa-miR-132	40.00	27.07	12.93
hsa-miR-224	40.00	27.69	12.31
hsa-miR-492	40.00	27.83	12.17
hsa-miR-28-3p	40.00	27.95	12.05
hsa-miR-628-3p	40.00	27.97	12.03
hsa-miR-769-5p	40.00	27.98	12.02
hsa-miR-758	40.00	27.98	12.02
hsa-miR-425#	40.00	27.99	12.01
hsa-miR-1267	40.00	28.01	11.99

hsa-miR-629	40.00	28.01	11.99
hsa-miR-485-3p	40.00	28.02	11.98
hsa-miR-223#	40.00	28.03	11.97
hsa-miR-199a	40.00	28.73	11.27
hsa-miR-636	40.00	28.85	11.15
hsa-miR-543	40.00	28.87	11.13
hsa-miR-591	40.00	28.87	11.13
hsa-miR-330	40.00	28.90	11.10
hsa-miR-652	40.00	28.94	11.06
hsa-miR-181a-2#	40.00	28.95	11.05
hsa-miR-516-3p	40.00	28.97	11.03
hsa-miR-1180	40.00	28.97	11.03
hsa-miR-1179	40.00	28.99	11.01
hsa-miR-1262	40.00	28.99	11.01
hsa-miR-151-5P	40.00	28.99	11.01
hsa-miR-199a-3p	40.00	29.00	11.00
hsa-miR-204	40.00	29.00	11.00
hsa-miR-330-5p	40.00	29.81	10.19
hsa-miR-320B	40.00	29.93	10.07
hsa-miR-200c	40.00	29.94	10.06
hsa-miR-16-2#	40.00	29.94	10.06
hsa-miR-572	40.00	29.94	10.06
hsa-miR-518b	40.00	29.94	10.06
hsa-miR-15b	40.00	29.95	10.05
hsa-miR-625	40.00	29.95	10.05
hsa-miR-95	40.00	29.95	10.05
hsa-miR-454#	40.00	29.96	10.04
hsa-miR-142-3p	40.00	30.01	9.99
hsa-miR-128a	40.00	30.03	9.97
hsa-miR-19b-1#	40.00	30.11	9.89
hsa-miR-518f	40.00	30.90	9.10
hsa-miR-454	35.04	25.94	9.09
hsa-miR-519b-3p	40.00	30.92	9.08
hsa-miR-10b#	40.00	30.96	9.04
hsa-miR-1291	40.00	30.96	9.04
hsa-miR-378	40.00	30.97	9.03
hsa-miR-361	40.00	30.97	9.03
hsa-miR-148a	40.00	30.98	9.02
hsa-miR-539	40.00	30.98	9.02
hsa-miR-99b#	40.00	31.00	9.00
hsa-miR-561	40.00	31.09	8.91
hsa-miR-627	40.00	31.89	8.11
hsa-miR-886-3p	40.00	31.91	8.09
hsa-miR-1269	40.00	31.92	8.08

hsa-miR-1227	40.00	31.93	8.07
hsa-miR-523	40.00	31.93	8.07
hsa-miR-191#	40.00	31.97	8.03
hsa-miR-1271	40.00	31.99	8.01
hsa-miR-1243	40.00	31.99	8.01
hsa-miR-942	40.00	32.00	8.00
hsa-miR-136	40.00	32.01	7.99
hsa-miR-488	40.00	32.01	7.99
hsa-miR-146b	35.96	27.98	7.97
hsa-miR-489	40.00	32.04	7.96
hsa-miR-362	40.00	32.16	7.84
hsa-miR-203	40.00	32.93	7.07
hsa-miR-744	40.00	32.94	7.06
hsa-miR-629	40.00	32.94	7.06
hsa-miR-1300	40.00	32.94	7.06
hsa-miR-196b	40.00	33.01	6.99
hsa-miR-888	40.00	33.02	6.98
hsa-miR-26b	27.07	20.96	6.10
hsa-miR-1276	40.00	33.91	6.09
hsa-miR-106b#	40.00	33.99	6.01
hsa-miR-339-5p	40.00	34.01	5.99
hsa-miR-144#	40.00	34.04	5.96
hsa-miR-548c-5p	40.00	34.09	5.91
hsa-miR-376a	36.06	30.94	5.12
hsa-miR-185	40.00	34.92	5.08
hsa-miR-517b	40.00	34.96	5.04
hsa-miR-23a#	40.00	34.97	5.03
hsa-miR-151-3p	29.97	24.95	5.02
hsa-miR-106b	25.97	31.03	-5.06
hsa-miR-1289	33.03	40.00	-6.97
hsa-miR-141#	26.00	33.06	-7.06
hsa-miR-340	32.00	40.00	-8.00
hsa-miR-324-5p	31.90	40.00	-8.10
hsa-miR-181a	31.85	40.00	-8.15
hsa-miR-202	16.91	25.98	-9.07
hsa-miR-548d	29.95	40.00	-10.05
hsa-miR-576-3p	28.97	40.00	-11.03
hsa-miR-381	21.97	33.01	-11.04
hsa-miR-548a	28.95	40.00	-11.05
hsa-miR-125b	28.06	40.00	-11.94
hsa-miR-10a	28.05	40.00	-11.95
hsa-miR-423-5p	27.00	40.00	-13.00
hsa-miR-194	14.70	28.01	-13.31
hsa-miR-520c-3p	15.96	29.87	-13.91

hsa-miR-302a	20.95	40.00	-19.05
hsa-miR-628-5p	8.97	32.98	-24.01
hsa-miR-651	13.28	40.00	-26.72
hsa-miR-302c	9.42	40.00	-30.58

Supplemental Data Table S3

Differentially expressed miRNAs in pre-operative CRC serum samples compared to post-operative CRC serum samples determined by TLDA.

miRNA	Ct of pre-operative CRC	Ct of post-operative CRC	-ΔCt (pre-operative – post-operative)
hsa-miR-1274B	19.88	40.00	20.12
hsa-miR-504	20.57	40.00	19.43
hsa-miR-382	22.79	40.00	17.21
hsa-miR-601	22.94	40.00	17.06
hsa-miR-1233	22.95	40.00	17.05
hsa-miR-484	22.95	40.00	17.05
hsa-miR-380-3p	22.98	40.00	17.02
hsa-let-7b	23.01	40.00	16.99
hsa-miR-654-3p	23.62	40.00	16.38
hsa-miR-1274A	23.93	40.00	16.07
hsa-miR-126#	23.98	40.00	16.02
hsa-let-7e	23.99	40.00	16.01
hsa-miR-145	24.00	40.00	16.00
hsa-miR-197	24.01	40.00	15.99
hsa-miR-409-5p	24.93	40.00	15.07
hsa-miR-151-3p	24.95	40.00	15.05
hsa-miR-331-5p	24.96	40.00	15.04
hsa-miR-30b	24.98	40.00	15.02
hsa-miR-766	25.00	40.00	15.00
hsa-miR-26a	25.01	40.00	14.99
hsa-miR-1260	25.04	40.00	14.96
hsa-miR-515-5p	25.72	40.00	14.28
hsa-miR-34b	25.80	40.00	14.20
hsa-miR-574-3p	25.91	40.00	14.09
hsa-miR-133a	25.97	40.00	14.03
hsa-miR-139-5p	26.00	40.00	14.00
hsa-miR-486	26.01	40.00	13.99
hsa-miR-1305	26.02	40.00	13.98
hsa-miR-139-3p	26.03	40.00	13.97
hsa-miR-30d	26.03	40.00	13.97
hsa-miR-1303	26.23	40.00	13.77
hsa-miR-672	26.29	40.00	13.71
hsa-miR-412	26.81	40.00	13.19
hsa-miR-218-1#	26.83	40.00	13.17

hsa-miR-532-3p	26.89	40.00	13.11
hsa-miR-221	26.94	40.00	13.06
hsa-miR-625#	26.95	40.00	13.05
hsa-miR-27a	26.97	40.00	13.03
hsa-miR-432	26.98	40.00	13.02
hsa-miR-193b	26.98	40.00	13.02
hsa-miR-125a-5p	26.98	40.00	13.02
hsa-miR-30a-3p	26.99	40.00	13.01
hsa-miR-331	27.00	40.00	13.00
hsa-miR-365	27.01	40.00	12.99
hsa-miR-409-3p	27.01	40.00	12.99
hsa-miR-1183	27.04	40.00	12.96
hsa-miR-132	27.07	40.00	12.93
hsa-miR-130a#	27.07	40.00	12.93
hsa-miR-224	27.69	40.00	12.31
hsa-miR-492	27.83	40.00	12.17
hsa-miR-505#	27.91	40.00	12.09
hsa-miR-324-3p	27.93	40.00	12.07
hsa-miR-28-3p	27.95	40.00	12.05
hsa-miR-206	27.95	40.00	12.05
hsa-miR-628-3p	27.97	40.00	12.03
hsa-miR-769-5p	27.98	40.00	12.02
hsa-let-7c	27.98	40.00	12.02
hsa-miR-146b	27.98	40.00	12.02
hsa-miR-758	27.98	40.00	12.02
hsa-miR-375	27.99	40.00	12.01
hsa-miR-425#	27.99	40.00	12.01
hsa-miR-1267	28.01	40.00	11.99
hsa-miR-629	28.01	40.00	11.99
hsa-miR-485-3p	28.02	40.00	11.98
hsa-miR-223#	28.03	40.00	11.97
hsa-miR-320	23.98	35.94	11.96
hsa-miR-638	28.14	40.00	11.86
hsa-miR-199a	28.73	40.00	11.27
hsa-miR-636	28.85	40.00	11.15
hsa-miR-543	28.87	40.00	11.13
hsa-miR-591	28.87	40.00	11.13
hsa-miR-330	28.90	40.00	11.10
hsa-miR-885-5p	28.92	40.00	11.08
hsa-miR-652	28.94	40.00	11.06
hsa-miR-181a-2#	28.95	40.00	11.05
hsa-miR-127	28.97	40.00	11.03
hsa-miR-1180	28.97	40.00	11.03
hsa-miR-100	28.98	40.00	11.02

hsa-miR-140-3p	28.98	40.00	11.02
hsa-miR-520D-3P	28.98	40.00	11.02
hsa-miR-1179	28.99	40.00	11.01
hsa-miR-1262	28.99	40.00	11.01
hsa-miR-151-5P	28.99	40.00	11.01
hsa-miR-199a-3p	29.00	40.00	11.00
hsa-miR-26b#	29.01	40.00	10.99
hsa-miR-330-5p	29.81	40.00	10.19
hsa-miR-720	21.93	32.03	10.10
hsa-miR-152	29.90	40.00	10.10
hsa-miR-886-5p	29.90	40.00	10.10
hsa-miR-320B	29.93	40.00	10.07
hsa-miR-328	29.93	40.00	10.07
hsa-miR-200c	29.94	40.00	10.06
hsa-miR-16-2#	29.94	40.00	10.06
hsa-miR-572	29.94	40.00	10.06
hsa-miR-518b	29.94	40.00	10.06
hsa-miR-15b	29.95	40.00	10.05
hsa-miR-95	29.95	40.00	10.05
hsa-miR-454#	29.96	40.00	10.04
hsa-miR-339-3p	29.96	40.00	10.04
hsa-miR-193a-5p	29.98	40.00	10.02
hsa-miR-323-3p	29.98	40.00	10.02
hsa-miR-642	30.00	40.00	10.00
hsa-miR-30c	23.99	33.99	10.00
hsa-miR-142-3p	30.01	40.00	9.99
hsa-miR-19b-1#	30.11	40.00	9.89
hsa-miR-494	30.91	40.00	9.09
hsa-miR-125a-3p	30.94	40.00	9.06
hsa-miR-376a	30.94	40.00	9.06
hsa-miR-27a#	30.95	40.00	9.05
hsa-miR-10b#	30.96	40.00	9.04
hsa-miR-1291	30.96	40.00	9.04
hsa-miR-378	30.97	40.00	9.03
hsa-miR-361	30.97	40.00	9.03
hsa-miR-148a	30.98	40.00	9.02
hsa-miR-539	30.98	40.00	9.02
hsa-miR-28	30.99	40.00	9.01
hsa-miR-296	30.99	40.00	9.01
hsa-miR-223	16.96	25.97	9.01
hsa-miR-99b#	31.00	40.00	9.00
hsa-miR-1290	17.96	26.95	8.99
hsa-miR-483-5p	23.97	32.92	8.95
hsa-miR-335	31.05	40.00	8.95

hsa-miR-561	31.09	40.00	8.91
hsa-miR-195	24.01	32.89	8.87
hsa-miR-337-5p	15.74	23.87	8.14
hsa-miR-545	31.88	40.00	8.12
hsa-miR-627	31.89	40.00	8.11
hsa-miR-93#	31.91	40.00	8.09
hsa-miR-886-3p	31.91	40.00	8.09
hsa-miR-1269	31.92	40.00	8.08
hsa-miR-1227	31.93	40.00	8.07
hsa-miR-523	31.93	40.00	8.07
hsa-miR-191#	31.97	40.00	8.03
hsa-miR-218	31.97	40.00	8.03
hsa-miR-342-3p	23.94	31.95	8.01
hsa-miR-1271	31.99	40.00	8.01
hsa-miR-942	32.00	40.00	8.00
hsa-miR-511	32.00	40.00	8.00
hsa-miR-422a	32.01	40.00	7.99
hsa-miR-136	32.01	40.00	7.99
hsa-miR-488	32.01	40.00	7.99
hsa-miR-92a	25.01	33.00	7.98
hsa-miR-18a#	32.03	40.00	7.97
hsa-miR-489	32.04	40.00	7.96
hsa-miR-345	27.98	35.93	7.96
hsa-miR-362	32.16	40.00	7.84
hsa-miR-30a-5p	24.94	32.02	7.08
hsa-miR-203	32.93	40.00	7.07
hsa-miR-744	32.94	40.00	7.06
hsa-miR-629	32.94	40.00	7.06
hsa-miR-1300	32.94	40.00	7.06
hsa-miR-645	32.96	40.00	7.04
hsa-miR-20b	29.00	36.04	7.04
hsa-miR-372	32.96	40.00	7.04
hsa-miR-122	24.96	31.99	7.03
hsa-miR-597	32.98	40.00	7.02
hsa-miR-99b	26.98	34.00	7.02
hsa-miR-184	32.99	40.00	7.01
hsa-miR-222	22.98	29.99	7.01
hsa-miR-150	21.00	28.00	7.00
hsa-miR-502-3p	33.00	40.00	7.00
hsa-miR-381	33.01	40.00	6.99
hsa-miR-196b	33.01	40.00	6.99
hsa-miR-888	33.02	40.00	6.98
hsa-miR-24	20.00	26.97	6.96
hsa-miR-141#	33.06	40.00	6.94

hsa-miR-191	19.98	26.89	6.91
hsa-miR-182	30.95	37.06	6.11
hsa-miR-1276	33.91	40.00	6.09
hsa-miR-454	25.94	31.97	6.02
hsa-miR-216a	18.86	24.88	6.02
hsa-miR-106b#	33.99	40.00	6.01
hsa-miR-339-5p	34.01	40.00	5.99
hsa-miR-27b	28.01	34.00	5.99
hsa-miR-214	26.00	31.96	5.96
hsa-miR-144#	34.04	40.00	5.96
hsa-miR-1255B	28.93	34.04	5.12
hsa-miR-185	34.92	40.00	5.08
hsa-miR-329	34.93	40.00	5.07
hsa-miR-517b	34.96	40.00	5.04
hsa-miR-106a	21.96	26.99	5.03
hsa-miR-23a#	34.97	40.00	5.03
hsa-miR-17	21.95	26.98	5.03
hsa-miR-26b	20.96	25.98	5.02
hsa-miR-548b-5p	40.00	34.00	-6.00
hsa-miR-1254	40.00	33.98	-6.02
hsa-miR-548d	40.00	33.94	-6.06
hsa-miR-519d	40.00	33.93	-6.07
hsa-miR-618	30.00	23.91	-6.09
hsa-miR-548d-5p	40.00	33.02	-6.98
hsa-miR-576-3p	40.00	33.00	-7.00
hsa-miR-190	35.99	28.98	-7.02
hsa-miR-590-5p	40.00	32.95	-7.05
hsa-miR-1	40.00	31.97	-8.03
hsa-miR-647	40.00	31.93	-8.07
hsa-miR-1184	40.00	31.81	-8.19
hsa-miR-340	40.00	30.96	-9.04
hsa-miR-517a	40.00	30.93	-9.07
hsa-miR-708	40.00	30.73	-9.27
hsa-miR-216b	40.00	30.05	-9.95
hsa-miR-548a	40.00	29.87	-10.13
hsa-miR-181a	40.00	29.10	-10.90
hsa-miR-518d	40.00	29.02	-10.98
hsa-miR-615-5p	40.00	28.88	-11.12

Supplemental Data Table S4

Differentially expressed miRNAs both in pre-operative CRC serum samples compared to post-operative CRC serum samples and pre-operative CRC serum samples compared to post-operative CRC serum samples determined by TLDA.

miRNA	Ct of healthy control	Ct of pre-operative CRC	Ct of post-operative CRC	-ΔCt (pre-operative - control)	-ΔCt (pre-operative– post-operative)
hsa-miR-216a	40	18.86	24.88	21.14	6.02
hsa-miR-193a-3p	40	19.79	40	20.21	16.38
hsa-miR-504	40	20.57	40	19.43	19.43
hsa-miR-106a	40	21.96	26.99	18.04	5.03
hsa-miR-382	40	22.79	40	17.21	17.21
hsa-miR-17	40	22.97	26.98	17.03	5.03
hsa-miR-214	40	26	31.96	14	5.96
hsa-miR-139-3p	40	26.03	40	13.97	13.97
hsa-miR-412	40	26.81	40	13.19	13.19
hsa-miR-199a-3p	40	29	40.00	11	11.00
hsa-miR-572	40	29.94	40	10.06	10.06
hsa-miR-518b	40	29.94	40	10.06	10.06
hsa-miR-15b	40	29.95	40	10.05	10.05
hsa-miR-95	40	29.95	40	10.05	9.02
hsa-miR-627	40	31.89	40	8.11	8.11
hsa-miR-146b	35.96	27.98	40	7.97	12.02
hsa-miR-26b	27.07	20.96	25.98	6.1	5.02
hsa-miR-517b	40	34.96	40	5.04	5.04

Supplemental Data Table S5

Differentially expressed miRNAs by comparing pre-operative, postoperative CRC serum samples to healthy control serum samples validated by quantitative RT-PCR[1].

miRNA	Healthy control	Pre-operative CRC	Post-operative CRC	*P* value[2]	*P* value[3]	Result
miR-106a	0.30 ± 0.009	0.44± 0.015	0.29 ± 0.009	<0.0001	<0.0001	significant
miR-17-3p	0.011 ± 0.0005	0.019 ± 0.0008	0.012 ± 0.0004	<0.0001	<0.0001	significant
miR-145	0.20± 0.007	0.13± 0.004	0.19 ± 0.005	<0.0001	<0.0001	Significant
miR-15b	0.14± 0.012	0.15±0.009	0.017±0.01	0.22	0.70	non-significant
miR-17	0.15± 0.016	0.14±0.012	0.17±0.023	0.42	0.66	non-significant
miR-21	0.25± 0.006	0.31±0.005	0.32±0.006	0.31	0.37	non-significant
miR-26b	0.95±0.11	1.07± 0.125	0.95± 0.143	0.53	0.47	non-significant
miR-31	0.95 ± 0.062	0.94 ± 0.064	0.93 ± 0.135	0.95	0.88	non-significant
miR-139-3p	0.52 ±0.014	0.68±0.023	0.78±0.03	0.38	0.27	non-significant
miR-143	0.56 ± 0.03	0.51 ± 0.02	0.52 ± 0.01	0.35	0.83	non-significant
miR-146b	1.07 ±0.13	1.41±0.14	1.13±0.13	0.6	0.08	non-significant
miR-214	0.35 ±0.04	0.32±0.03	0.42±0.03	0.02	0.84	non-significant
miR-216a	0.31 ±0.06	0.28±0.04	0.36±0.06	0.44	0.66	non-significant
miR-193a-3p	0.39 ± 0.060	0.37 ± 0.032	0.44± 0.033	0.06	0.39	non-significant
miR-199a-3p	0.16±0.02	0.16±0.03	0.19±0.04	0.55	0.54	non-significant
miR-382	0.26 ±0.06	0.58±0.05	0.57±0.04	0.001	0.38	non-significant
miR-412						detection rate < 75%
miR-517b						detection

	rate < 75%
miR-572	detection rate < 75%
miR-518b	Ct value > 35
miR-504	Ct value > 35
miR-627	Ct value > 35

[1]The relative contents of miRNAs are presented as mean±SEM.

[2] Difference between pre-operative CRC and post-operative CRC.

[3] Difference between pre-operative CRC and post-operative CRC.

Figure S1

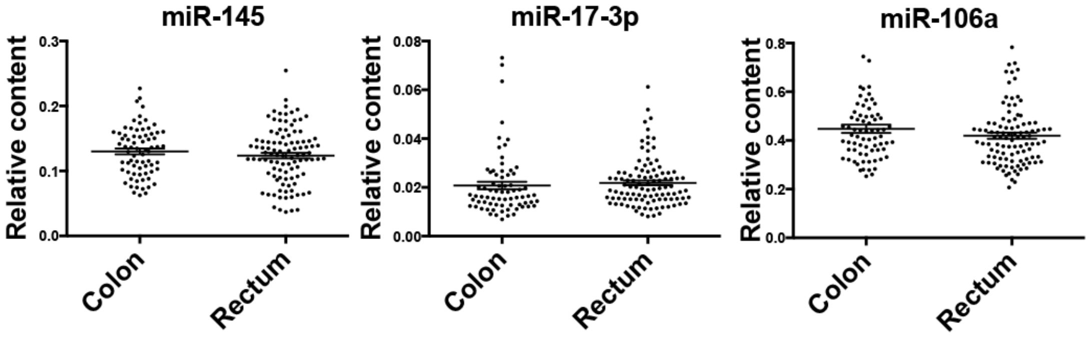
miR-145
miR-17-3p
miR-106a
Relative content
0.3
0.2
0.1
0.0
0.08
0.06
0.04
0.02
0.00
0.8
0.6
0.4
0.2
0.0
Colon
Rectum
